# Healthy and Sustainable Food Choices in European Households: A Cross-Cultural Study of Knowledge, Practices, Intentions, and Barriers

**DOI:** 10.3390/nu18162689

**Published:** 2026-08-18

**Authors:** Ana B. Baranda, Noelia da Quinta, Patricia Rioja, Yolanda Ríos, Jelena M. Meinilä, Kamilla Bargiel-Matusiewicz, Natalia Ziółkowska, Maria E. Heikkilä, Mayke Kramer, Maijaliisa Erkkola, Elena Santa Cruz

**Affiliations:** 1 AZTI, Food Research, Basque Research and Technological Alliance (BRTA), Parque Tecnológico de Bizkaia, Astondo Bidea, Edificio 609, 48160 Derio, Bizkaia, Spain; ndaquinta@azti.es (N.d.Q.); prioja@azti.es (P.R.); yrios@azti.es (Y.R.); esantacruz@azti.es (E.S.C.); 2 Department of Food and Nutrition, University of Helsinki, 00790 Helsinki, Finland; jelena.meinila@helsinki.fi (J.M.M.); maria.e.heikkila@helsinki.fi (M.E.H.); mayke.kramer@helsinki.fi (M.K.); maijaliisa.erkkola@helsinki.fi (M.E.); 3 Faculty of Psychology, University of Warsaw, 02-097 Warsaw, Poland; kmatusiewicz@psych.uw.edu.pl (K.B.-M.); natalia.ziolkowska@psych.uw.edu.pl (N.Z.)

**Keywords:** food choices, healthy eating, sustainable diets, dietary behaviors, dietary barriers, households with children, cross-cultural study

## Abstract

**Background:** Growing concerns about health and sustainability are accelerating the need to transform food systems to promote well-being and reduce environmental impacts. Despite increasing policy efforts, evidence remains limited on self-perceived knowledge, reported practices, intentions, and perceived barriers related to healthy and sustainable eating among European households with children. Establishing healthy eating habits during childhood is essential for long-term health, with parents playing a pivotal role in shaping these behaviors. Understanding the factors and barriers related to food choices is essential for informing interventions that support healthier and more sustainable dietary practices. **Methods:** This study, conducted in 2023 as part of the European TITAN project, explored parental perceptions and practices related to healthy and sustainable eating in Spain, Poland, and Finland. A structured questionnaire was completed by 240 parents of children aged 6–12 in each country using quota sampling based on national income tertiles. **Results:** Although households reported high self-perceived knowledge, patterns consistent with a possible discrepancy between self-perceived knowledge and reported food choices were observed. Finnish households, despite showing the highest recognition of predefined healthy and sustainable eating concepts, reported lower self-reported adherence to healthy and sustainable practices. Reducing meat consumption and choosing sustainably sourced seafood were among the least valued actions across all countries. Cost was the most frequently reported barrier, especially in Finland (60%), alongside greater difficulty in forming new habits and resisting less healthy options. Overall, cost and behavioral constraints were among the most frequently reported barriers to healthier and more sustainable dietary practices. **Conclusions:** These findings highlight the need for culturally tailored strategies to support households in transitioning toward healthier and more sustainable dietary behaviors. Addressing perceived barriers and food-choice constraints may be important for supporting healthier and more sustainable dietary practices at the household level.

## 1. Introduction

The global spread of Western dietary patterns has exerted severe pressure on both human health, driving non-communicable diseases [1], and the environment, accelerating ecosystem degradation and greenhouse gas emissions [2]. Consequently, transitioning to sustainable healthy diets, a holistic approach rooted in the One Health perspective, which recognizes the interconnectedness of human, animal, and environmental well-being, is a pressing global challenge [3,4]. According to the FAO and WHO, sustainable healthy diets are dietary patterns that promote health and well-being while exerting low environmental impacts and are accessible, affordable, culturally acceptable, nutritionally adequate, safe, and healthy [5].

Transforming the food system requires actions across the entire supply chain, where food choices and consumer behaviour represent critical dimensions [6,7]. At the consumer level, these diets emphasize plant-based foods, minimize red meat and ultra-processed products, and promote sustainable practices such as reducing food waste and prioritizing local, minimally packaged goods [5].

Previous studies have reported possible discrepancies between knowledge-related indicators and dietary practices among consumers. While awareness of certain sustainability aspects (e.g., packaging waste) is growing, the environmental impact of meat production and the practicalities of shifting to plant-based diets remain less frequently recognised, and dietary transition is hindered by motivational barriers [8,9]. Furthermore, even well-known guidelines, such as adequate fruit and vegetable intake, suffer from low adherence across Europe [10]. These observations highlight the need to better understand the factors shaping food choices and their relationship with adherence to healthy and sustainable diets.

Behavioural theories may help interpret these discrepancies. The COM-B model proposes that behaviour results from the interaction between capability, opportunity, and motivation [11]. Within this framework, knowledge contributes to psychological capability, whereas economic, environmental, and behavioural barriers may constrain opportunity and motivation. Thus, positive intentions may not necessarily be accompanied by corresponding dietary practices, helping to contextualize difficulties in adopting healthy and sustainable diets. For example, a study in Finland found that transitioning to plant-based diets, particularly those with a 70:30 plant-to-animal protein ratio, requires additional support to ensure nutritional adequacy and consumer adherence [12], highlighting the importance of exploring consumer readiness across countries.

Ages 6–12 years represent a key developmental period for the establishment of dietary habits that may persist into adulthood [13]. During these years, children progressively develop greater autonomy in food preferences and eating behaviours, while parents continue to shape food availability, meal planning, and dietary routines [6,7,14,15]. This makes households with school-aged children a relevant setting for examining how parental knowledge, intentions, perceived barriers, and reported practices relate to healthy and sustainable eating.

Although several European studies have examined consumer perceptions of healthy and sustainable diets, food choice motivations, or barriers to dietary change, these dimensions are often investigated separately [16,17]. Evidence focusing specifically on parents and households with school-aged children remains comparatively limited, particularly regarding how self-perceived knowledge, concept recognition, intentions, perceived barriers, and self-reported practices coexist across different cultural contexts.

To address this gap, we conducted a cross-cultural study in 2023 to describe and compare parental self-perceived knowledge, recognition of concepts related to healthy and sustainable diets, self-reported dietary practices, future intentions, and perceived barriers among households with children across three contrasting European contexts.

Spain, Finland, and Poland were selected because they represent contrasting European contexts in terms of dietary traditions, socioeconomic conditions, sustainability performance, school meal policy coverage, and public nutrition infrastructures. Spain provides a Mediterranean dietary context, Finland a Nordic food environment with strong institutional nutrition support, and Poland a Central European context with different levels of policy integration regarding sustainable diets (Table 1). A total of 720 households with children aged 6–12 years were surveyed across the three countries. This diversity enables an exploratory cross-cultural comparison to inform targeted interventions for household-level dietary shifts. The study forms part of the European TITAN project, specifically the case study “Pilot on 360° Healthy Nutritional Habits for Children” (website: https://titanproject.eu/), which aims to support households with children in adopting healthier and more sustainable dietary patterns.

## 2. Materials and Methods

### 2.1. Sample and Study Design

We conducted a cross-sectional online survey in 2023 among 720 households with children aged 6–12 years in Spain, Finland, and Poland (*n* = 240/country). Participants were adults (≥18 years) primarily or jointly responsible for household food purchasing and planning, who responded on behalf of their households. Sample size was determined within the framework of the TITAN project to enable descriptive cross-country comparisons while maintaining balanced representation across income tertiles in each participating country. No formal a priori power calculation was conducted.

A non-probability quota sampling approach was used to obtain equal representation across three country-specific household income tertiles (low, medium, high; *n* = 80/tertile/country), defined according to national statistical frameworks. Household income categories were based on country-specific household income thresholds and were not equivalised by household size. Although quotas were used to balance income groups and achieve broad geographical coverage, the resulting sample should not be considered nationally representative. No weighting procedures were applied, and the sample was not formally compared with national population distributions.

An international market research agency administered the culturally adapted and translated questionnaire (Spanish, Finnish, Polish) through an online platform, using established consumer panels and Nielsen’s segmentation to achieve broad geographical coverage across participating countries. Eligible respondents were adults (≥18 years) living in households with at least one child aged 6–12 years and primarily or jointly responsible for household food purchasing and meal planning. Survey access was restricted to one participation per respondent, and only complete questionnaires were included in the final dataset. Recruitment and quality-control procedures were managed by the market research agency. The study complied with the Declaration of Helsinki and ICC/ESOMAR codes. The Research Ethics Committee at the Faculty of Psychology of the University of Warsaw approved the protocols (opinion nos. 1/06/2023 and 2/06/2023) under the European TITAN project.

The 53-item questionnaire assessed dietary habits and food choice-related behaviors. This study analyzes variables related to: (1) self-perceived knowledge, (2) dietary adherence, (3) future intentions, and (4) barriers to healthy and sustainable eating (Table 2). Items were evaluated using 5-point Likert scales and multiple-choice questions, adapted from previous validated studies [23,24,25,26]. In line with the multidimensional FAO/WHO definition [5], sustainability-related items were used to capture household-level perceptions and practices associated with sustainable healthy diets, including affordability, cultural acceptability, food waste, packaging, seasonality, and protein sources. The questionnaire was developed by the AZTI research team using items adapted from previously published studies together with project-specific questions addressing dietary habits, food-related behaviors, knowledge, attitudes, and household practices. The questionnaire formed part of a broader survey, of which only the variables relevant to the present study are reported here. Content relevance and cultural suitability were reviewed by project partners from Finland and Poland. Country-specific adaptations were made where necessary, particularly for dietary guideline-related items and other questions requiring contextual adaptation to national circumstances.

Prior to data collection, we pre-tested the questionnaire with individuals matching the target population profile but not involved in the project. Feedback from this process was used to improve item clarity, evaluate comprehension, assess completion time, and reduce the risk of respondent fatigue.

To minimize response bias, items within multi-concept questions were randomized. Sociodemographic characteristics (Table 3) were collected to describe the participating households; however, the analyses focused exclusively on descriptive cross-country comparisons.

### 2.2. Statistical Analyses

We conducted data analysis using XLSTAT (version 2019.1.2). One-way ANOVA with Tukey’s post-hoc test was used to evaluate country effects on continuous variables. Pearson’s chi-square test was used to examine differences between countries for categorical variables. For multiple-choice items, each item was analysed separately by comparing selection and non-selection frequencies across countries. Although Likert-scale responses are ordinal in nature, they were analysed using parametric methods because of the relatively large sample size and balanced study design across countries [27]. Responses indicating high agreement (Likert scores of 4–5) were additionally expressed as percentages as descriptive indicators of high agreement or adherence. Statistical significance was set at *p* < 0.05. Analyses were descriptive and exploratory in nature and focused on cross-country comparisons. Effect sizes, confidence intervals, and multivariable adjustment procedures were not estimated.

## 3. Results

The final analytical dataset included 720 complete questionnaires, comprising 240 respondents from each participating country. Country comparisons for Likert-scale variables were based primarily on mean scores, whereas percentages of respondents scoring 4–5 are presented as descriptive indicators of high agreement or adherence. For multiple-choice items, percentages refer to the proportion of respondents in each country selecting each option.

### 3.1. Characteristics of the Participating Households

The main characteristics of participating households are presented in Table 3. Female respondents were more prevalent in Finland and Poland, whereas gender distribution was more balanced in Spain. Most respondents were between 35 and 44 years of age, and more than half reported a university degree or higher level of education. Household income quotas were equally distributed across countries by design.

### 3.2. Self-Perception and Knowledge of Healthy and Sustainable Diets

**Figure 1:**
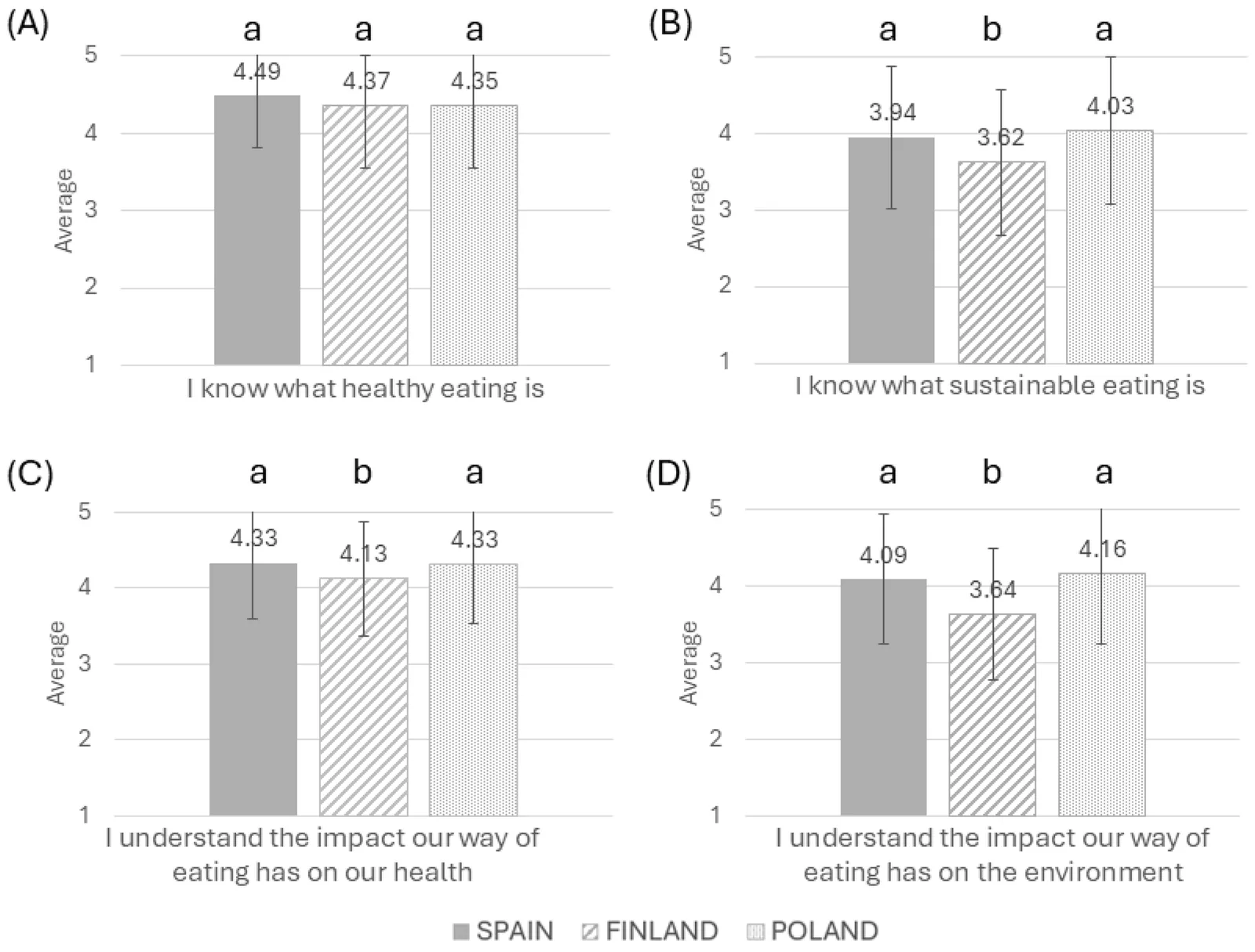
Household self-perceived knowledge (5-point scale) regarding healthy (**A**) and sustainable (**B**) diets, and the perceived impact of their diet on health (**C**) and the environment (**D**). Values are presented as mean ± SD; error bars represent standard deviations. Different letters indicate statistically significant differences between countries according to one-way ANOVA followed by Tukey’s post-hoc test (*p* < 0.05).

Self-perceived healthy eating knowledge was high across all countries (mean > 4.3; *p* = 0.100; Figure 1A), with 88.7% to 92.9% of households reporting high levels of agreement (Likert scores 4–5). Conversely, the perceived impact of diet on health was significantly lower in Finland (mean 4.1) than in Spain and Poland (both mean 4.3) (*p* < 0.001; Figure 1C).

Nevertheless, most households (>83%) understood the health implications of their diets. Conversely, self-perceived knowledge and understanding of dietary environmental impacts were significantly lower in Finland (mean 3.6; ~58–60% agreement) compared to Spain and Poland (>69% agreement) (*p* < 0.001). These results indicate cross-country differences in perceived knowledge, particularly regarding environmental aspects of diets.

**Figure 2:**
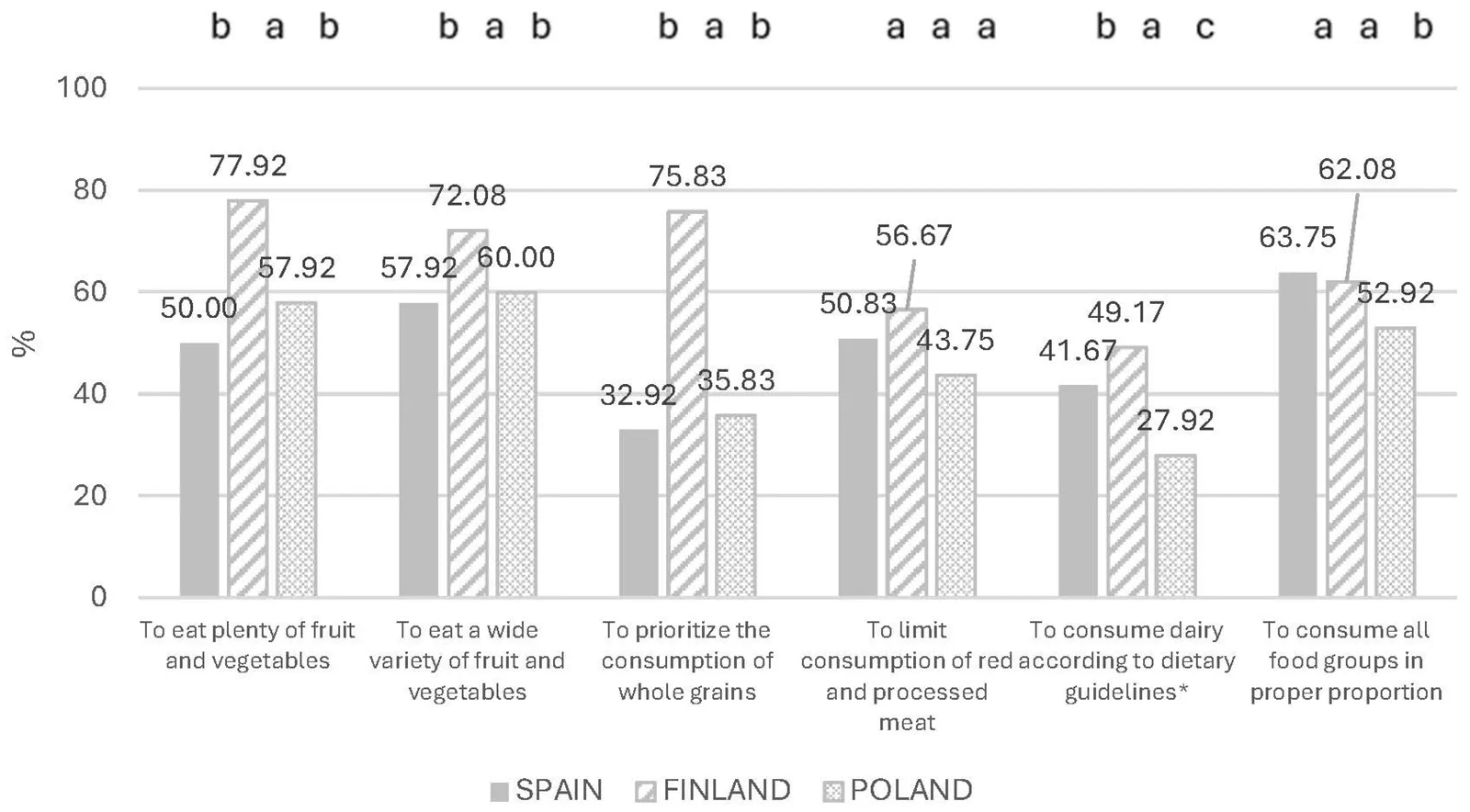
Proportion of households selecting healthy eating concepts. * See the footnote to Table 2 for details. Different letters indicate statistically significant differences in the proportion of respondents selecting each item between countries according to Pearson’s chi-square test for each multiple-choice item (*p* < 0.05). Percentages are calculated using 240 respondents per country; for each multiple-choice item, values represent the proportion of respondents selecting that option.

For healthy eating concept recognition (Table 2, Q2), Finnish households showed higher overall concept selection rates (~65%) than Spanish and Polish households (~46–50%) (Figure 2; Supplementary Table S1).

Over 70% of Finnish households prioritized “eating plenty/a wide variety of fruits and vegetables” and “prioritizing whole grains”. Notably, the selection rate for “prioritizing whole grains” was significantly lower in Spain (32.92%) and Poland (35.83%) (*p* < 0.001).

Meanwhile, limiting red and processed meat showed no significant differences across countries (43.75–56.67%; *p* = 0.188). However, Polish households reported significantly lower rates for “consuming all food groups in proper proportion” (52.92%) compared to Spain and Finland (~62–64%) (*p* < 0.05).

**Figure 3:**
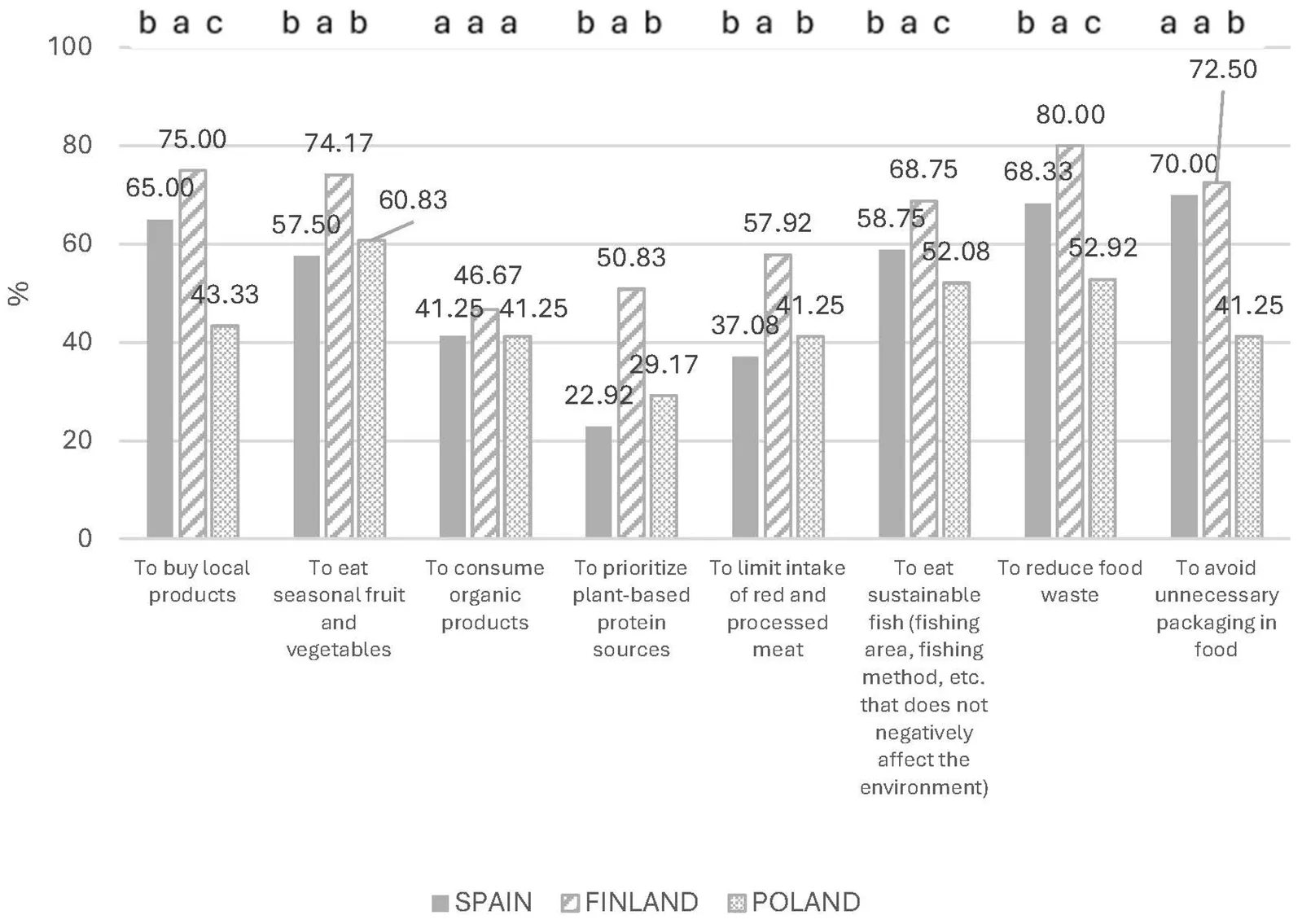
Proportion of households selecting sustainable eating concepts. Different letters indicate statistically significant differences in the proportion of respondents selecting each item between countries according to Pearson’s chi-square test for each multiple-choice item (*p* < 0.05). Percentages are calculated using 240 respondents per country; for each multiple-choice item, values represent the proportion of respondents selecting that option.

Regarding sustainable eating concept recognition (Table 2, Q3), significant differences were observed across countries for all items except “consuming organic products” (Figure 3; Supplementary Table S2).

Finnish households reported the highest number of sustainability-related concepts selected (mean number of concepts selected: 5.85), compared to Spain (4.65) and Poland (3.86). Spain and Finland most frequently selected “buying local and seasonal products”, “avoiding unnecessary packaging”, and “reducing food waste” (all *p* < 0.05). Conversely, “prioritizing plant-based proteins” and “limiting meat” were the least frequently selected concepts across all countries. “Eating sustainable fish” also showed significant cross-country differences (*p* = 0.037).

### 3.3. Adherence to Healthy and Sustainable Diets

**Figure 4:**
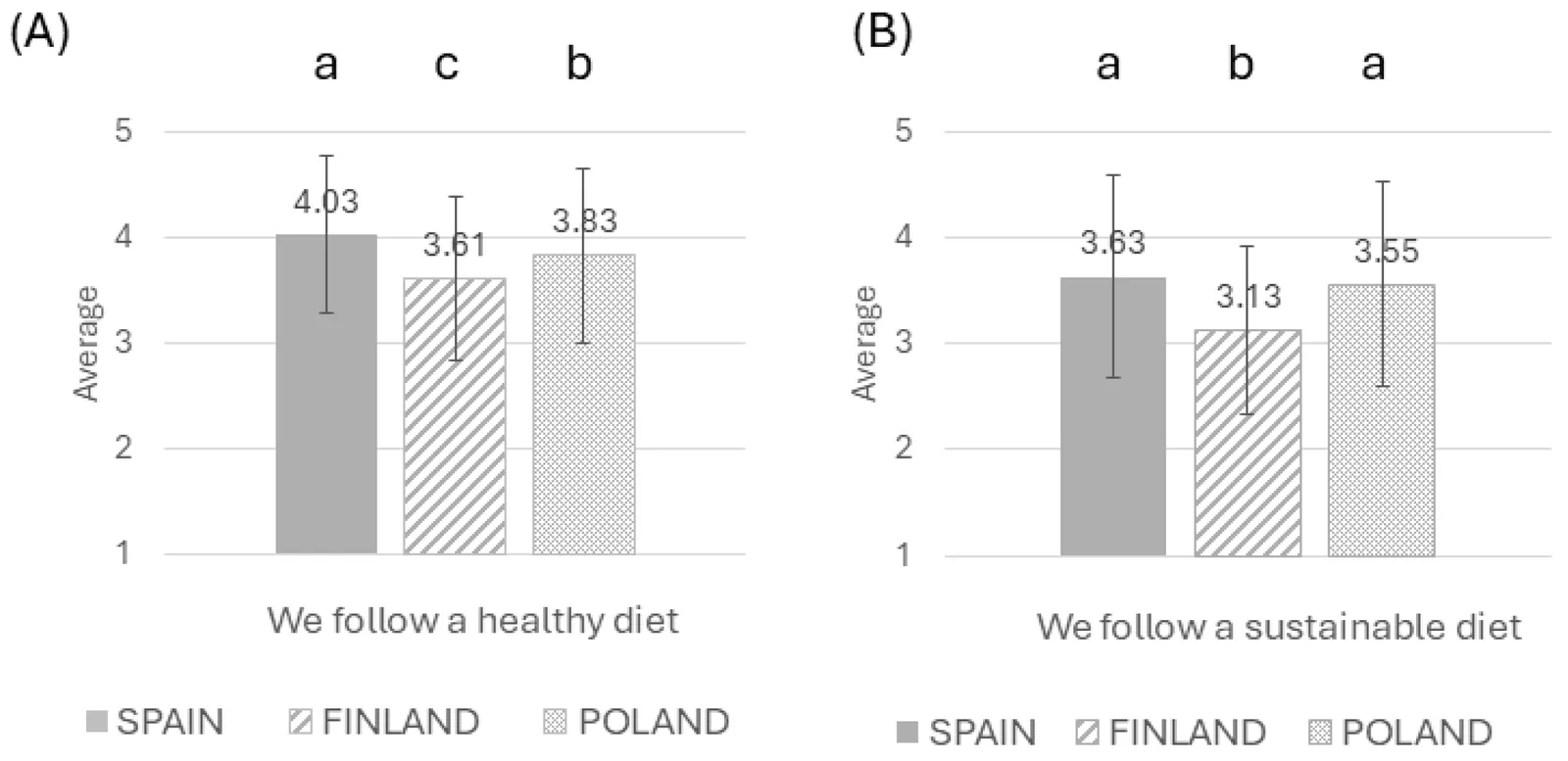
Self-perceived household adherence to a healthy (**A**) and sustainable (**B**) diet (5-point scale). Values are presented as mean ± SD; error bars represent standard deviations. Different letters indicate statistically significant differences between countries according to one-way ANOVA followed by Tukey’s post-hoc test (*p* < 0.05).

Spanish households reported the highest adherence to a healthy diet (mean 4.0), followed by Poland (3.8) and Finland (3.6) (*p* < 0.001; Figure 4A).

The proportion of households reporting high healthy diet adherence (scores 4–5) was greatest in Spain (~78%), followed by Poland (~69%) and Finland (~62%) (*p* < 0.05).

For sustainable diets (Figure 4B), Spain and Poland reported higher means (3.6) and comparable high adherence rates (~53–56%; *p* = 0.118), both higher than Finland (mean 3.1; ~27% high adherence; *p* < 0.001).

**Figure 5:**
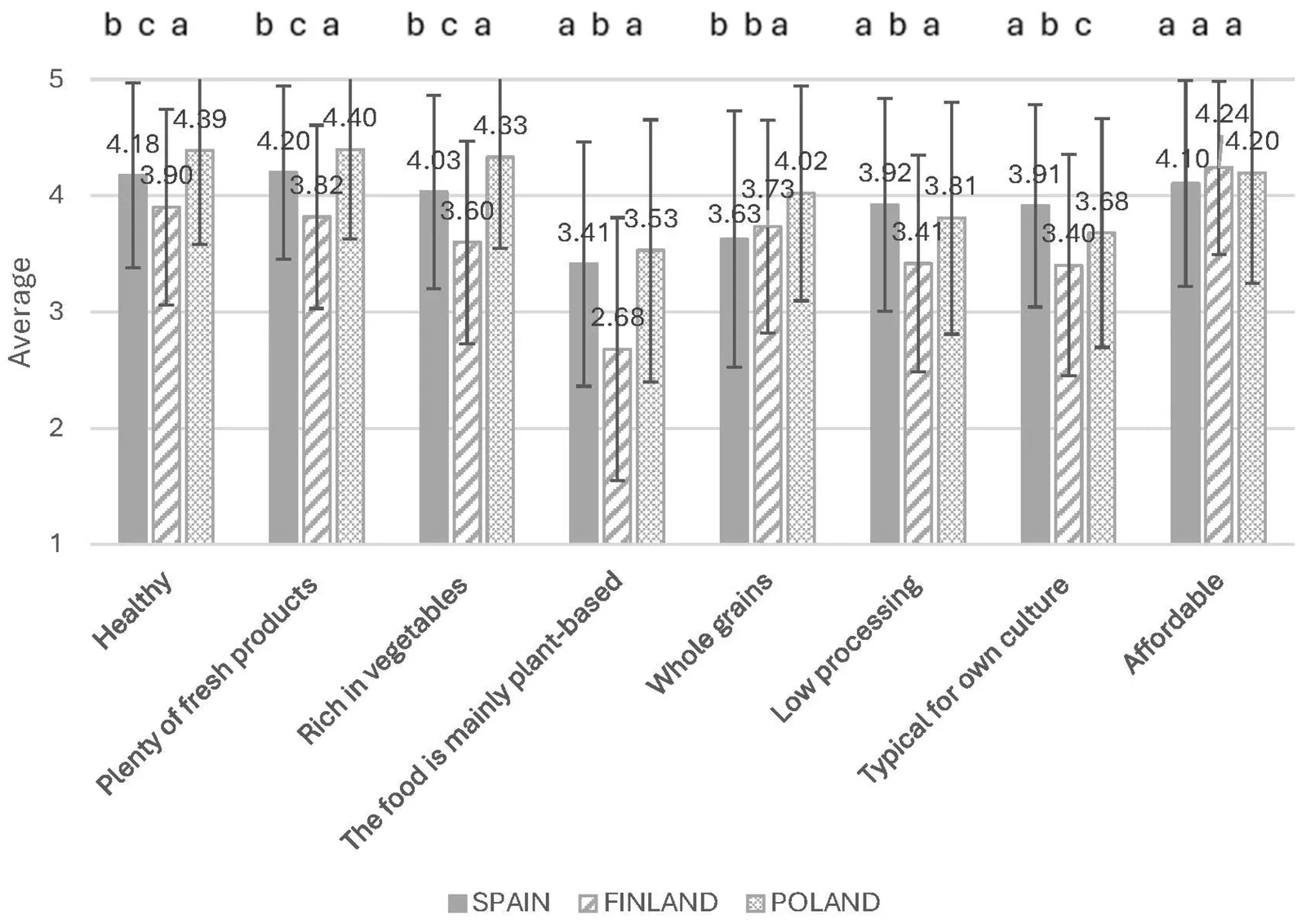
Household-rated importance of food characteristics during meal planning or shopping. Values are presented as mean ± SD; error bars represent standard deviations. Different letters indicate statistically significant differences between countries according to one-way ANOVA followed by Tukey’s post-hoc test (*p* < 0.05).

Regarding food characteristics during meal planning/shopping (Table 2, Q5), all items showed significant cross-country differences except affordability (*p* = 0.220), which was universally rated highly (Figure 5).

The concepts “healthy” and “plenty of fresh food” were highly rated overall, though significant differences emerged (both *p* < 0.001). Polish households reported higher mean scores (4.4 for both) and high importance ratings (~88%), followed closely by Spain (~83%), whereas Finland reported the lowest scores (means 3.8–3.9; ~71% high importance).

Being “rich in vegetables” was highly valued in Poland and Spain (means >4.0; ~73–87% high importance), with significantly higher ratings than in Finland, where only ~58% of households rated it as important (*p* < 0.001). Importance of “whole grains” was highest in Poland (~73%), followed by Finland (~64%) and Spain (~58%) (*p* < 0.05). Regarding “low processing” and “cultural typicality,” Finnish households reported significantly lower scores (mean 3.4; ~49% importance) than Spanish and Polish households (~60% importance; *p* < 0.001). The item “the food is mainly plant-based” was the least valued food characteristic overall. While households in Spain and Poland assigned moderate importance to this characteristic (~48–55%), Finnish respondents assigned lower importance, with a mean score of 2.7 and only 24% high importance (*p* < 0.001).

**Figure 6:**
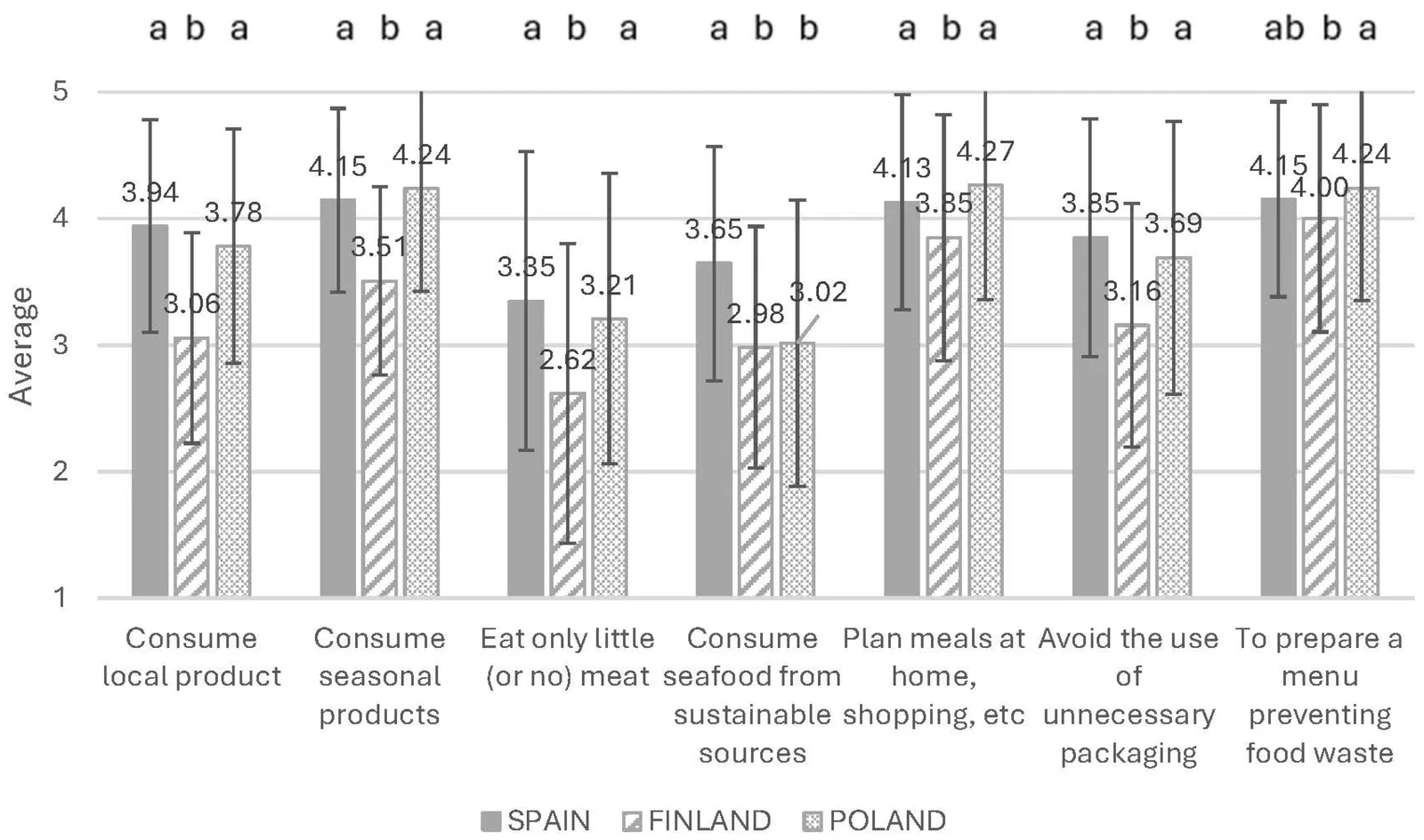
Household adherence to sustainable behaviours (5-point scale). Values are presented as mean ± SD; error bars represent standard deviations. Different letters indicate statistically significant differences between countries according to one-way ANOVA followed by Tukey’s post-hoc test (*p* < 0.05).

Adherence to specific sustainable behaviours (Table 2, Q6) varied significantly across countries for most items (*p* < 0.001; Figure 6). Only “preparing a menu to prevent food waste” averaged ≥4.0 in all countries.

Finnish households reported lower self-reported adherence to most sustainable behaviours, including “local and seasonal consumption” and “waste-reduction planning” (all *p* < 0.001). High adherence (scores 4–5) to “consuming local products” and “avoiding unnecessary packaging” was significantly higher in Spain and Poland (~61–70%) than in Finland (~27–33%). Across all countries, the behaviours with the lowest reported adherence were “eating little/no meat” (~23–46%; *p* = 0.02) and “consuming sustainable seafood,” with Spain reporting a significantly higher proportion of high adherence (~55%) than Poland and Finland (~30–33%; *p* < 0.001).

### 3.4. Future Intention Towards Healthy and Sustainable Diets

**Figure 7:**
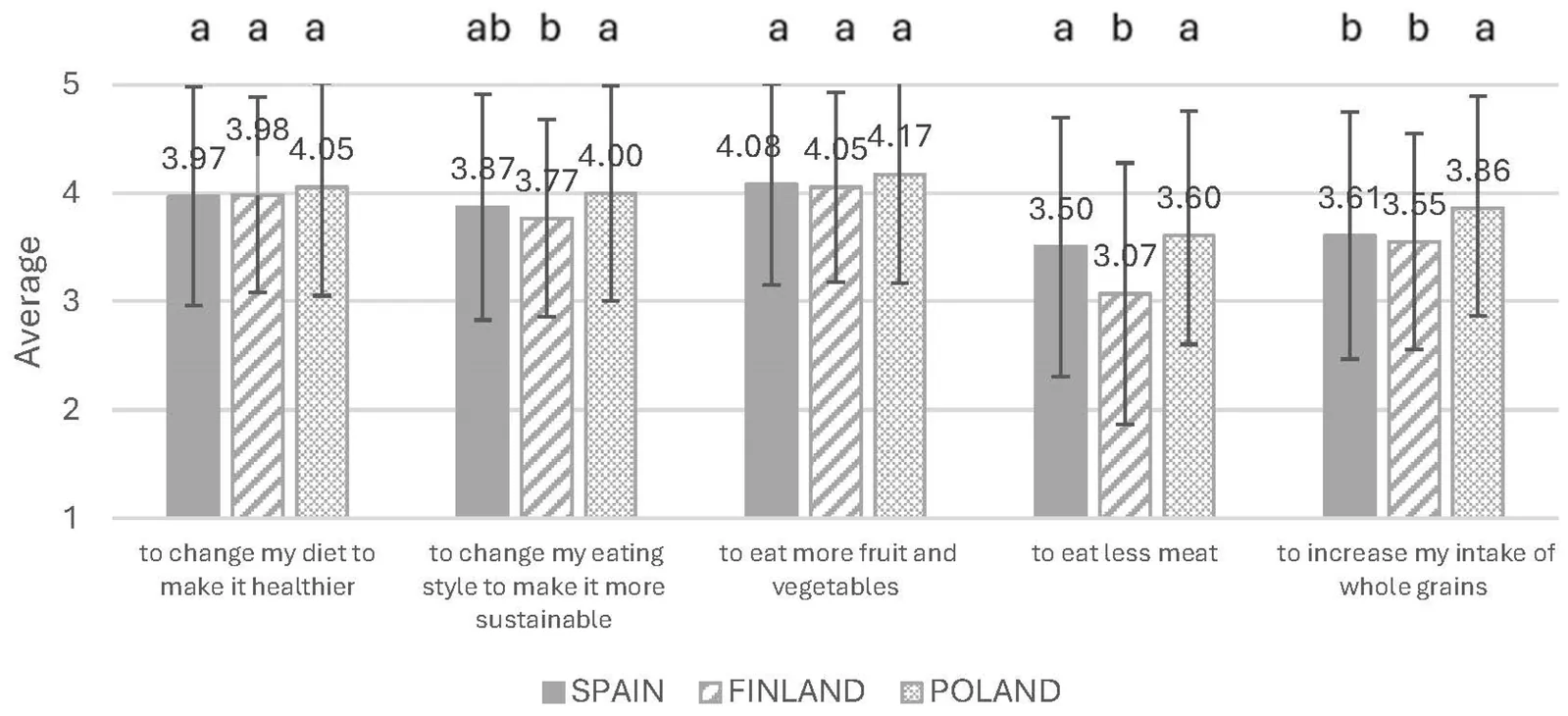
Household intention (5-point scale) toward healthy and sustainable dietary changes (statements phrased: “I would like to…”). Values are presented as mean ± SD; error bars represent standard deviations. Different letters indicate statistically significant differences between countries according to one-way ANOVA followed by Tukey’s post-hoc test (*p* < 0.05).

Parents showed a positive intention to improve dietary patterns (mean ~4.0; Figure 7), though scores were slightly lower for sustainability, which varied by country (*p* = 0.024). Overall, most parents reported high intention (scores 4–5) to shift toward healthier (~75%) and more sustainable (~69%) diets.

Intention to increase fruit and vegetable consumption was high and uniform across countries (*p* = 0.240). However, intention to reduce meat consumption was significantly lower in Finland (~38% high agreement) compared to Spain and Poland (~54–55%; *p* < 0.001). Conversely, intention to increase whole grain intake was highest among Polish households (mean 3.9; ~65% high agreement) compared to Spain and Finland (means 3.6; ~55–58% high agreement; *p* < 0.01).

### 3.5. Barriers to Adopting a Healthy and Sustainable Diet

**Figure 8:**
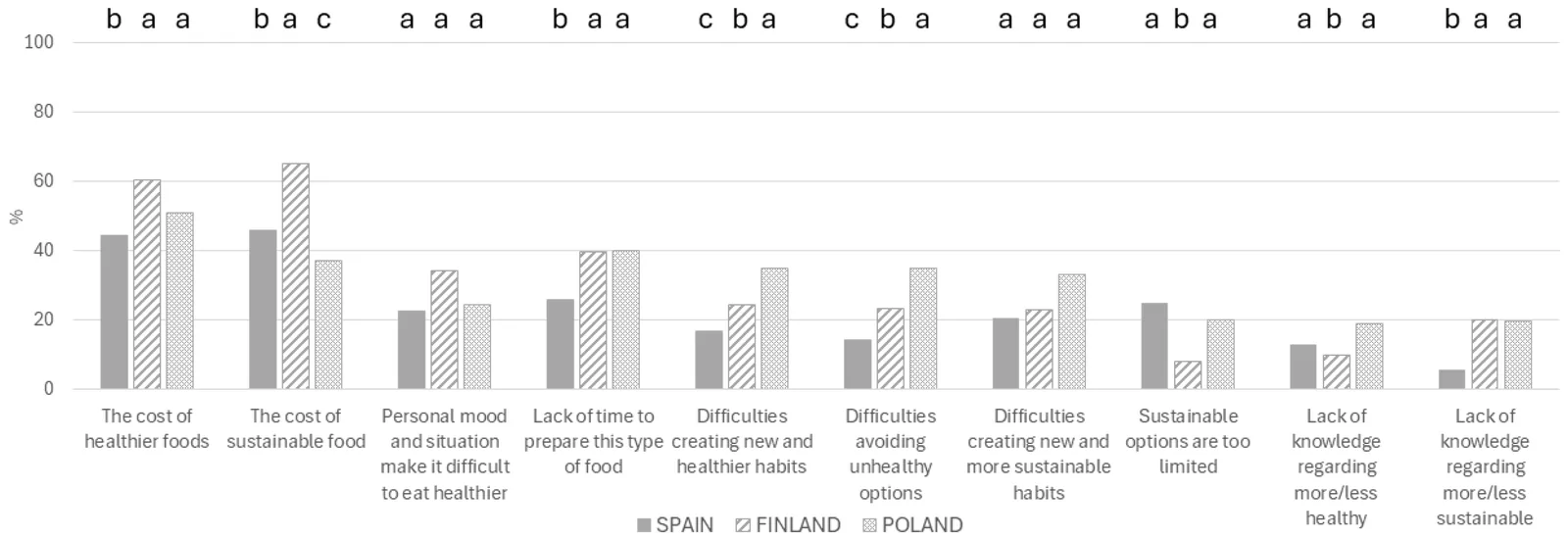
Household barriers to healthy and sustainable eating. Low-relevance items (<14% selection) are omitted. Different letters indicate statistically significant differences in the proportion of respondents selecting each barrier between countries according to Pearson’s chi-square test for each multiple-choice item (*p* < 0.05). Percentages are calculated using 240 respondents per country; for each multiple-choice barrier, values represent the proportion of respondents selecting that option. Complete *n* (%) values for all barriers are provided in Supplementary Table S3.

Most barriers were infrequently selected (Figure 8, Supplementary Table S3), except for food cost. “The cost of healthy food” was a barrier for ~44–60% of households, with Spain reporting the lowest rate (*p* < 0.05). “The cost of sustainable food” showed wider variation (*p* < 0.001), ranging from 65% in Finland to ~37% in Poland.

After cost, “personal mood/situation” was the second most cited barrier across countries (*p* = 0.132). Among households intending to improve their diet, this barrier was cited by ~32%, while cost reached ~61%. “Lack of time” was more frequently reported in Finland and Poland (~39%), but significantly less so in Spain (~26%; *p* < 0.01). Households in Finland were twice as likely to report greater difficulty in creating new habits and avoiding less healthy options (~35% vs. ~15% in Spain and Poland; *p* < 0.05).

Regarding sustainability, “creating new habits” was a common challenge (~20–33%), especially for those willing to change (~22%, alongside ~61% citing cost). Spanish and Polish households felt “sustainable options are too limited” significantly more than households in Finland (~20–25% vs. ~8%; *p* = 0.004). Knowledge-based barriers were generally low (<20%); however, lacking sustainable dietary knowledge was more prevalent in Poland and Finland (~20%) than in Spain (~5%; *p* = 0.004).

Finally, fewer than 14% of households reported no barriers, and only <10% expressed a lack of interest in healthier or more sustainable eating (*p* > 0.05).

## 4. Discussion

This study examined parental perceptions, habits, and intentions regarding healthy and sustainable eating among the surveyed households across three culturally and socioeconomically diverse European countries: Spain, Poland, and Finland. Overall, the findings indicate that respondent parents generally reported high self-perceived knowledge of healthy eating, but recognition of specific healthy and sustainable dietary concepts and self-reported practices varied across countries. The results also suggest a possible discrepancy between self-perceived knowledge and reported food choices, as higher self-perceived knowledge and positive intentions were not consistently accompanied by healthier or more sustainable self-reported dietary behaviors.

Practical and behavioural barriers, particularly food cost, time constraints, and difficulties establishing new habits, appeared more prominent than lack of interest or lack of knowledge, while meat reduction and plant-based protein-related behaviours remained among the weakest sustainability-related practices.

It is important to acknowledge that considerable heterogeneity exists within each country. Therefore, references throughout this manuscript to Spanish, Finnish, or Polish households are intended as shorthand descriptions of the sampled groups included in the study rather than as representations of homogeneous national populations.

While previous research has often focused on isolated aspects of sustainable diets, this is, to our knowledge, the first study to provide a comprehensive cross-cultural assessment of self-perceived knowledge, concept recognition, intentions, perceived barriers, and self-reported dietary practices among households with school-aged children.

### 4.1. Self-Perception and Knowledge of Healthy and Sustainable Diets

Across all three countries, households reported high self-perceived knowledge of healthy eating, yet self-perceived knowledge of sustainable diets was considerably lower, consistent with previous findings [28,29]. This discrepancy may be relevant when interpreting differences in food choices and adherence to sustainable dietary practices. Several predefined dietary concepts were not consistently recognised, mainly among Spanish and Polish households.

In contrast, Finnish households reported greater recognition of practices aligned with healthy and sustainable guidelines, likely reflecting long-standing national policies that integrate nutrition education into schools and workplaces [30]. Conversely, Polish households showed the lowest recognition of predefined concepts, a disparity potentially linked to the country’s lower standing in EU sustainability rankings compared to Finland [31].

Misconceptions regarding sustainable diets remain widely documented [32,33]. These may reflect a disconnect between abstract sustainability principles and the personal values or well-being goals that guide consumer behaviour. As argued by other authors [34], sustainable consumption is more likely to be adopted when it aligns with the pursuit of personal fulfilment. This misalignment may contribute to suboptimal food choices despite high self-perceived knowledge [34]. Notably, Spanish households were the least likely to identify “eating plenty of fruit and vegetables” as a healthy practice, despite Mediterranean traditions and “5-a-day” initiatives [35].

Furthermore, “limiting red and processed meat” was seldom associated with health, particularly in Poland, despite consumers in all three countries generally exceeding recommended intake limits [36,37,38]. Reducing red meat was also rarely recognized as a sustainable practice, echoing previous findings [9,39].

Other dietary components showed varying levels of recognition. Dairy was among the least selected behaviours, potentially due to rising health concerns and shifting preferences toward plant-based alternatives [40]. Whole grain consumption was more strongly associated with health by Finnish households, reflecting effective public health campaigns [41] and the widespread consumption of rye and porridge [42] compared to lower awareness in Spain and Poland [43].

Similarly, “balanced food proportions” was better recognized in Finland, though it did not exceed 62% in any country, highlighting how interpretations of this cornerstone concept vary across populations [28,44].

Regarding sustainability, Spanish and Finnish households most widely recognized food waste reduction, local/seasonal products, and packaging avoidance, as shown in previous studies [45,46]. However, nearly half of Polish parents did not identify waste reduction as a sustainable practice, consistent with prior reports [47]. This limited recognition in Poland of waste, packaging, and local foods may reflect broader consumer confusion, likely due to the absence of official definitions and a general misunderstanding of environmental implications, consistent with prior findings [48,49,50].

Finally, plant-based proteins were less frequently recognised as components of sustainable diets, as also reported in other studies [32,51], with only a minority of parents (23–51%) selecting them as a sustainable choice. Organic products were also not widely prioritised by families. Often promoted as sustainable, their consumption is often driven by perceived health benefits rather than environmental altruism [50,52]. Overall, these findings suggest that improving knowledge alone may be insufficient to support changes in food choices without clearer alignment between dietary guidance and consumer understanding.

### 4.2. Adherence to Healthy and Sustainable Diets

An apparent discrepancy remains between reported knowledge and self-reported behaviour, as suggested here and in previous research [33,53], with households across all countries prioritizing health over sustainability [32,54]. This discrepancy may reflect a misalignment between knowledge and food choices that could limit adherence to healthy and sustainable dietary patterns. Although participants appeared to place greater emphasis on health-related than sustainability-related dietary aspects, the present study was not designed to directly evaluate whether health and environmental motivations were perceived as complementary or competing influences on food choice. Future research should further explore how these motivations interact and whether aligning health and sustainability messages could support dietary change.

Spanish and Polish households emphasized “healthy” products during planning, yet actual adherence to national guidelines remains low, often limited by inadequate whole grain and dairy intake alongside excessive red meat consumption [55,56]. Interestingly, Finnish households, despite their higher recognition of predefined healthy and sustainable dietary concepts, assigned lower importance to several healthy and sustainability-related food characteristics during meal planning. This apparent discrepancy may reflect the influence of factors beyond knowledge alone. Previous research suggests that economic considerations, contextual constraints, and behavioural factors may shape food choices and influence the extent to which nutritional knowledge is translated into dietary practices [57]. In the present study, cost was the most frequently reported barrier among Finnish households, which may be consistent with this interpretation. Affordability is recognised as a critical dimension of sustainable diets [58]; however, the descriptive design of the present study did not allow us to determine whether affordability or other contextual factors influenced food choices or accounted for the observed discrepancy, as individual-level relationships and the mechanisms linking knowledge, food choices, and reported behaviour were not examined. These explanations should therefore be regarded as hypotheses for future research rather than mechanisms demonstrated by the present study.

Cultural and environmental factors may influence dietary emphasis. Although fresh products are a cornerstone of dietary guidelines [7], Finnish parents placed less importance on them, and on local or seasonal foods, compared to Spanish and Polish respondents. Finland’s cold climate and short growing seasons may restrict year-round local availability and foster greater reliance on preserved or frozen foods, which are often more affordable and convenient [59]. This environmental constraint may help explain the lower value Finnish households assign to being “rich in vegetables” and their traditional culinary emphasis on fish [41]. Conversely, Spain’s climate may support an abundant supply of fresh produce, reflected in Mediterranean culinary traditions and average shopping baskets [17,60]. Culinary heritage may also help explain why Polish households prioritized whole grains (rye, oats, buckwheat) notably present in traditional breads like chleb żytni [61], despite whole grain consumption remaining generally low across Europe [43].

Regarding protein sources, households across all countries did not place significant importance on adopting predominantly plant-based diets or eating little to no meat. Although Western Europe has seen a general decline in meat intake [62] and flexitarianism is rising [63], countries like Spain continue to report high consumption [64]. The transition toward reduced meat intake is more evident in intentions than in actual behaviour [44]. Many consumers may remain hesitant to reduce meat due to its cultural significance and strong sensory appeal [65], meaning traditional meat-centered cultures may continue to shape food choices [9,66]. Furthermore, cultural preferences may shape family dynamics; parents may need to navigate the tension between their sustainable dietary intentions and their children’s demand for familiar, culturally palatable foods like meat [67]. Consequently, plant-based options may remain marginal in Finland, where only 8% of consumers identify them as their primary protein source [68].

Other sustainable practices showed mixed adherence. Interestingly, despite historical reliance on processed options in some regions, parents across all three countries rated convenience foods lower than fresh options, reflecting a recent market reversal [69]. However, the low importance assigned to sustainable fish consumption likely reflects a broader unfamiliarity with certifications and traceability [70,71]. Finally, respondents across all countries prioritized household routines like “planning meals” and “preventing food waste,” recognized as effective waste-reduction strategies [26]. Yet, these behaviours are often driven by economic rather than environmental motives [72], as food constitutes a substantial budget share for families with children, who also generate significant waste [70,73]. Conversely, “avoiding unnecessary packaging” received low importance, supporting evidence that consumers prioritize price and quality over packaging sustainability [74].

### 4.3. Future Intentions to Make Dietary Changes

Households across all countries expressed positive intentions to adopt healthier and more sustainable dietary patterns, aligning with broader consumer trends [32,75]. A common priority was increasing fruit and vegetable consumption, reflecting growing European awareness of the health and environmental benefits of plant-based diets [76,77].

In contrast, parents showed limited motivation to reduce meat consumption, which may be related to the aforementioned cultural and familial barriers [9,78,79]. This possible resistance may also be reflected in a low perceived need for change, as shown by our findings that only 43–50% of Spanish and Polish households even associated meat reduction with healthy behaviour.

Regarding whole grains, households expressed a positive intention to increase intake, consistent with parental motivation to foster long-term healthy habits in children [80]. While this finding may be consistent with the greater recognition of whole grain consumption observed among Finnish households, the motivation in Spain and Poland remains unclear, as a substantial proportion of parents in these countries did not initially link whole grain consumption with healthy eating.

This pattern may be consistent with a possible discrepancy between concept recognition and reported intentions, although this relationship was not directly examined in the present study.

### 4.4. Barriers to Healthy and Sustainable Eating

Consumers face diverse challenges when adopting and maintaining sustainable diets, with practical, behavioral, and emotional barriers often outweighing informational ones [28,78]. Cultural values and social norms further shape these behaviors, suggesting that “one-size-fits-all” guidelines are less effective than tailored, culturally acceptable approaches [81,82].

In our study, cost emerged as the primary concern, especially in Finland, where climatic limitations may necessitate importing sustainable products, potentially increasing retail prices [83]. In contrast, Spain and Poland may benefit from contextual factors that support access to healthy and sustainable food options, including greater availability of locally produced foods [84].

This aligns with extensive evidence that the high cost of healthy and sustainable options remains a universal barrier, frequently driving households toward cheaper, less nutritious alternatives [78,85,86,87]. These economic constraints may strongly influence food choices and limit adherence to healthy and sustainable diets.

Lack of time, identified as a key barrier particularly among Finnish and Polish households, is consistently reported in the literature as a major impediment to prioritizing healthy eating and meal planning [88]. This constraint may result in a heavier reliance on convenience foods, which tend to be less healthy and sustainable [89].

Beyond logistical constraints, “personal mood or situation” was frequently cited as a barrier across all three countries, suggesting that emotional factors and stress may remain relevant even when nutritional knowledge is high [90]. This may reflect a broader “behavioural inertia” [91], particularly among younger cohorts who may struggle to resist the widespread availability of affordable, unhealthy options amidst daily demands [78,92].

Although cited by fewer than 20% of households, perceived or reported gaps in nutritional and sustainability knowledge may still hinder informed decision-making, as reported in other studies [33,88]. In our study, many participants reported high self-perceived knowledge but did not consistently recognise all predefined concepts, suggesting that misconceptions may remain, as previously reported in the literature [86]. Furthermore, as observed elsewhere, even high nutritional literacy is often overridden by family habits, time, and emotional states [75,91]. These findings are consistent with a possible discrepancy between self-perceived knowledge and reported food choices in real-life settings.

### 4.5. Study Limitations and Implications for Future Research

This study has several limitations. First, the cross-sectional design precludes conclusions regarding causality or the direction of associations between knowledge, intentions, barriers, and dietary behaviours. Moreover, the study did not directly examine individual-level associations among knowledge-related indicators, intentions, perceived barriers, and self-reported practices.

In addition, the study relied on self-reported responses, which may be subject to social desirability bias. Although parents answered on behalf of the household, the results primarily reflect individual perceptions rather than complex intra-household negotiations. Recruitment through an online consumer panel may have introduced selection bias, as participation was limited to individuals willing and able to complete online surveys.

The inclusion of households with children across a relatively broad age range (6–12 years) may have introduced variability in how children influence food purchasing and dietary decisions, as food-related autonomy can differ substantially across developmental stages.

The study used a non-probability quota sampling approach, and no weighting procedures were applied. Consequently, the findings should not be interpreted as nationally representative, but rather as reflecting the surveyed households included in the study samples. Furthermore, the analyses were descriptive and unadjusted, and sociodemographic variables such as gender, education, and income were not included as analytical variables. Therefore, some observed differences between countries may partly reflect differences in sample composition, including educational level, dietary pattern, health status, food allergies or intolerances, city size, and other household characteristics. In addition, country-level comparisons cannot capture the full diversity that exists within each national context and should therefore be interpreted as differences observed among the sampled households rather than definitive national characteristics.

The analytical approach was designed to provide descriptive cross-country comparisons and did not include effect size estimates, confidence intervals, multivariable adjustment procedures, or formal corrections for multiple testing. The sample size was also not based on a formal a priori power calculation. Accordingly, non-significant findings should also be interpreted with caution. Given the relatively large sample size, statistically significant differences should also be interpreted with caution, as they may not always reflect meaningful differences. Future studies should incorporate adjusted analyses, effect size measures, confidence intervals, and formal power calculations to strengthen the interpretation of cross-country differences.

Finally, because all variables were collected through the same self-reported questionnaire, common-method bias cannot be ruled out. Although the questionnaire was culturally adapted and pre-tested, subtle cross-country differences in item interpretation or response-scale use may have influenced comparisons. Formal measurement-invariance testing was not conducted. In addition, the study did not include objective measures of dietary intake, objective knowledge assessments, or direct indicators of the environmental impact of household food choices.

Despite these limitations, the study also presents several strengths. It included a relatively large sample of households from three European countries representing diverse dietary, socioeconomic, and policy contexts. The balanced allocation across income tertiles enhanced comparability between country samples, while the simultaneous assessment of self-perceived knowledge, intentions, barriers, and reported dietary behaviours provided a comprehensive perspective on factors related to healthy and sustainable food choices. Furthermore, the focus on households with children addresses an important and comparatively understudied population group. Future research should explore how sociodemographic, economic, and contextual factors interact to shape household food choices and dietary behaviours using longitudinal and multivariable approaches.

## 5. Conclusions

This study provides a cross-cultural perspective on self-reported knowledge, intentions, barriers, and dietary behaviors among households with children in Spain, Finland, and Poland. The findings reveal a landscape of high self-perceived health knowledge and positive intentions toward dietary improvement; however, high self-perceived knowledge and positive intentions coexisted with lower self-reported adherence to some healthier and more sustainable dietary practices. These findings are consistent with a possible discrepancy between self-perceived knowledge and reported food choices in real-life settings.

Sustainable practices, such as prioritizing plant-based proteins and reducing meat intake, were among the least recognized and least frequently adopted across all three countries. This underscores the need for nutrition education and consumer communication to further support the adoption of healthy and sustainable dietary behaviors. For dietetic practice, interventions could promote concrete, relatable behaviors, such as waste reduction or seasonal sourcing, that align with everyday household routines and cultural traditions.

Despite widespread intentions to change, practical, economic, and behavioral barriers remain important. Cost emerged as the most frequently reported obstacle, while time constraints and difficulties establishing new habits were also commonly reported barriers to dietary change. These findings suggest that knowledge and positive intentions alone may be insufficient to support dietary change.

Consequently, dietetic strategies must address not only informational needs but also the practical and motivational factors that shape daily choices and may constrain the adoption of healthy and sustainable dietary practices.

Cultural context is central to these dynamics. Dietary changes are embedded in local traditions and social norms; therefore, interventions must be culturally sensitive, adapting plant-based options to local tastes and supporting realistic food choices within everyday contexts.

Taken together, these findings suggest several practical implications for supporting households with children. Interventions could combine culturally tailored guidance with affordable meal ideas, simple weekly meal-planning tools, and strategies to facilitate healthier household routines. The gradual and culturally acceptable substitution of meat with affordable plant-based protein sources, particularly legumes, may support meat-reduction efforts, while clearer guidance on sustainably sourced seafood could facilitate more informed choices. At a broader level, measures aimed at improving the availability and affordability of healthy and sustainable foods may help households translate positive intentions into everyday dietary practices.

Finally, future longitudinal and intervention studies are needed to better understand how structural, economic, household, and behavioral factors relate to dietary behaviors and how targeted strategies can support healthier and more sustainable dietary patterns.

## Figures and Tables

**Table 1:** Comparative indicators for country selection.

Indicator	Spain	Finland	Poland	Source
Gross National Income per capita (USD)	~34,000	~56,000	~20,000	[16]
Dominant dietary model	Mediterranean ^1^	Nordic ^2^	Central European ^3^	^1^ [17] ^2^ [18] ^3^ [19]
Sustainability ranking (EU SDG Index)	12th	1st	24th	[20]
School meal policy coverage (%)	60	100	45	[21]
National dietary guidelines include sustainability	Yes	Yes	No	[22]

^1^ Mediterranean dietary model [17]. ^2^ Nordic dietary model [18]. ^3^ Central European dietary model [19].

**Table 2:** Questions and answers analysed for this paper.

Theme: Self-Perceived Knowledge of Healthy and Sustainable Diets	
Question 1	To what extent do you agree with the following statements?
Items	1-I know what healthy eating is
	2-I understand the impact our way of eating has on our health
	3-I know what sustainable eating is
	4-I understand the impact our way of eating has on the environment
Response	5-point scale labelled as 1 = strongly disagree, 2 = disagree, 3 = neutral, 4 = agree and 5 = strongly agree.
Question 2	Of the following concepts, which ones do you think are linked to healthy eating? Select all options that apply.
Items	1-To eat plenty of fruit and vegetables
	2-To eat a wide variety of fruit and vegetables
	3-To prioritize the consumption of whole grains
	4-To limit consumption of red and processed meat
	5-To consume dairy according to dietary guidelines *
	6-To consume all food groups in proper proportion
Response	Multiple choice.
Question 3	Of the following concepts, which ones do you think are linked to sustainable eating? Select all options that apply.
Items	1-To buy local products
	2-To eat seasonal fruit and vegetables
	3-To consume organic products
	4-To prioritize plant-based protein sources
	5-To limit intake of red and processed meat
	6-To eat sustainable fish (fishing area, fishing method, etc. that does not negatively affect the environment)
	7-To reduce food waste
	8-To avoid unnecessary packaging in food
Response	Multiple choice.
Theme: Adherence to healthy and sustainable diets	
Question 4	Considering the way of eating that your household follows, indicate your degree of agreement with the following statements.
Items	1-We follow a healthy diet
	2-We follow a sustainable diet
Response	5-point scale labelled as 1 = strongly disagree, 2 = disagree, 3 = neutral, 4 = agree and 5 = strongly agree.
Question 5	Indicate how important the following concepts are to you when planning meals at home, shopping, etc.
Items	1-Healthy
	2-Plenty of fresh products
	3-Rich in vegetables
	4-The food is mainly plant-based
	5-Whole foods (e.g., whole grains, such as barley rice, buckwheat, whole wheat bread, rye bread, whole wheat pasta)
	6-Low processing (avoid prepared foods that can be frozen or stored at room temperature, such as frozen foods, cooked foods, canned foods, and instant foods)
	7-Typical for own culture
	8-Affordable
Response	5-point scale labelled as 1 = never, 2 = almost never, 3 = occasionally, 4 = almost every day and 5 = always.
Question 6	To what extent does your household…?
Items	1-Consume local products, that is, it is marketed in the same territory where it is produced
	2-Consume seasonal products
	3-Consume seafood from sustainable sources (fishing area, fishing method, etc. that do not negatively affect the environment)
	4-Eat only little (or no) meat
	5-Plan meals at home, shopping, etc.
	6-Avoid the use of unnecessary packaging (e.g., fruit in plastic, meat/fish in trays, etc.)
	7-Prepare a menu preventing food waste (e.g., priority use of foods that are likely to go bad to ensure there are no leftovers)
Response	5-point scale labelled as 1 = never, 2 = almost never, 3 = occasionally, 4 = almost every day and 5 = always
Theme:	Future Intention towards healthy and sustainable diets
Question 7	To what extent do you agree with the following statements:
Items	1-I would like to change my diet to make it healthier
	2-I would like to change my eating style to make it more sustainable or respectful with the environment
	3-I would like to eat more fruit and vegetables
	4-I would like to increase my intake of whole grains
	5-I would like to eat less meat
Response	5-point scale labelled as 1 = strongly disagree, 2 = disagree, 3 = neutral, 4 = agree and 5 = strongly agree.
Theme	Barriers to adopting a healthy and sustainable diet
Question 8	What prevents you from eating more healthily and sustainably right now? Select all options that apply.
Items	1-The cost of healthier foods
	2-The cost of sustainable food
	3-Personal mood and situation make it difficult to eat healthier, e.g., unhealthy foods help me/us to relax, to deal with stress or to feel good
	4-Lack of time to prepare this type of food
	5-Difficulties creating new and healthier habits
	6-Difficulties avoiding unhealthy options
	7-Difficulties creating new and more sustainable habits
	8-Sustainable options are too limited
	9-Lack of knowledge regarding what is more or less healthy
	10-Lack of knowledge regarding what is more or less sustainable
	11-No interest in eating healthier
	12-Nothing, we already follow a healthy and sustainable diet
	13-No interest in eating taking into account the protection of the environment
	14-I don’t know
Response	Multiple choice.

* Country-specific dietary guidelines included in the questionnaire: Spain: maximum 2–3 servings of dairy per day (1 serving = a glass of milk or 2 yogurts). Finland: 5–6 dL of low-fat liquid dairy products and a few slices of cheese daily (adult recommendations). Poland: 2–3 servings of dairy per day, preferably low-fat or semi-skimmed options.

**Table 3:** Characteristics of the households participating in the study.

Households: 240 per Country with Children Aged from 6 to 12 Years; (*N* = 720)					
		Mean (%)			Total *n*
		Spain	Finland	Poland	
Gender adults	Male	50.8	36.7	24.6	269
	Female	48.3	62.9	74.6	446
	Non-binary gender	0.4	0	0.8	3
	I prefer not to answer	0.4	0	0	2
Age of adults (years)	25–34	111	43	57	211
	35–44	104	116	126	346
	45–54	23	70	49	142
	55–64	2	10	8	20
	>65	0	1	0	1
Educational level	Without studies or primary school	2.1	2.9	13.3	44
	Secondary school or intermediate and advanced vocational training	34.6	44.6	33.7	271
	First or second cycle university degree or higher (doctorate, master, …)	63.3	52.5	52.9	405
Employment situation	Housework	5.4	5	11.7	53
	Student	0.4	3.3	0.8	11
	Employee	85.4	75	77.5	571
	Unemployed	7	8.8	5	52
	Retired	0	1.7	0.4	5
	Unable to work owing to sickness or disability	0	3.8	2.1	15
	Other	0.4	2.5	2.5	13
Household income	Low *	33.3	33.3	33.3	240
	Medium *	33.3	33.3	33.3	240
	High *	33.3	33.3	33.3	240
City size	<10,000 inhabitants	11.7	16.3	20.4	116
	10,000–100,000 inhabitants	33.8	49.6	35.8	286
	100,000–1,000,000 inhabitants	28.8	23.8	26.7	190
	>1,000,000 inhabitants	25.8	10.4	17.1	128
Adults’ dietary patterns	Consumption of animal and plant-based foods (omnivorous)	69.3	91.2	66.2	544
	Mainly consumption of plant-based foods and a minor consumption of animal foods (flexitarian)	17.0	4.2	19.2	97
	Consumption of animal and plant-based foods, except meat (pescatarian)	5.8	2.5	7.5	38
	Consumption of plant-based products, dairy and eggs, but not meat and fish (vegetarian)	5.4	1.7	5.0	29
	Only consumption of plant-based products (vegan)	2.5	0.4	2.1	12
Person responsible for meal planning and with the greatest influence on eating behaviour at home	Respondent	63.8	52.9	63.3	432
	Respondent’s partner	5.4	7.5	8.8	52
	Both	30.8	39.6	27.9	236
Special factors influencing food choice	None	71.3	54.6	57.9	441
	Special diet (e.g., low-carbohydrate diet)	13.8	12.9	15	100
	Health condition (e.g., cardiovascular disease, diabetes, etc.)	13.3	17.5	22.1	127
	Food allergies and/or intolerances	11.7	30.4	17.9	144

* Household income: Low (Spain: <1600 €; Poland: <4000 zł; Finland: <2200 €); Medium (Spain: 1600–4400 €; Poland: 4000–7600 zł; Finland: 2200–5100 €); High (Spain: >4400 €; Poland: >7600 zł; Finland: >5100 €).

## Data Availability

The datasets generated and/or analyzed during the current study are available from the corresponding author on reasonable request.

## Supplementary Materials

https://www.mdpi.com/article/10.3390/nu18162689/s1, Table S1. Healthy Eating Concept Recognition (Question 2); Table S2. Sustainable Eating Concept Recognition (Question 3). Table S3. Barriers to Adopting a Healthy and Sustainable Diet (Question 8).
