# Supplementary material for: Healthy and Sustainable Food Choices in European Households: A Cross-Cultural Study of Knowledge, Practices, Intentions, and Barriers
Source: Nutrients. 2026 Aug 18;18(16):2689. doi: 10.3390/nu18162689 (PMC13516926; doi:10.3390/nu18162689)
Supplement: Supplementary file 1 [file nutrients-18-02689-s001.zip › nutrients-4398847-supplementary.pdf]

Supplementary Materials

Table S1. Healthy Eating Concept Recognition (Question 2)

Item	SPAIN n (%)	FINLAND n (%)	POLAND n (%)
**To eat plenty of fruit and vegetables**	120 (50.00)	187 (77.92)	139 (57.92)
**To eat a wide variety of fruit and vegetables**	139 (57.92)	173 (72.08)	144 (60.00)
**To prioritize the consumption of whole grains**	79 (32.92)	182 (75.83)	86 (35.83)
**To limit consumption of red and processed meat**	122 (50.83)	136 (56.67)	105 (43.75)
**To consume dairy according to dietary guidelines***	100 (41.67)	118 (49.17)	67 (27.92)
**To consume all food groups in proper proportion**	153 (63.75)	149 (62.08)	127 (52.92)

Note. Values are presented as n (%), based on 240 respondents per country. *Country-specific dietary guidelines included in the questionnaire are described in Table 2.

Table S2. Sustainable Eating Concept Recognition (Question 3)

Item	SPAIN n (%)	FINLAND n (%)	POLAND n (%)
**To buy local products**	156 (65.00)	180 (75.00)	104 (43.33)
**To eat seasonal fruit and vegetables**	138 (57.50)	178 (74.17)	146 (60.83)
**To consume organic products**	99 (41.25)	112 (46.67)	99 (41.25)
**To prioritize plant-based protein sources**	55 (22.92)	122 (50.83)	70 (29.17)
**To limit intake or red and processed meat**	89 (37.08)	139 (57.92)	99 (41.25)
**To eat sustainable fish**	141 (58.75)	165 (68.75)	125 (52.08)
**To reduce food waste**	164 (68.33)	192 (80.00)	127 (52.92)
**To avoid unnecessary packaging in food**	168 (70.00)	174 (72.50)	99 (41.25)

Note. Values are presented as n (%), based on 240 respondents per country.

Table S3. Barriers to Adopting a Healthy and Sustainable Diet (Question 8)

Barrier	SPAIN n (%)	FINLAND n (%)	POLAND n (%)
**The cost of healthier foods**	106 (44.17)	145 (60.42)	122 (50.83)
**The cost of sustainable food**	110 (45.83)	156 (65.00)	89 (37.08)
**Personal mood and situation make it difficult to eat healthier**	54 (22.50)	82 (34.17)	58 (24.17)
**Lack of time to prepare this type of food**	62 (25.83)	95 (39.58)	96 (40.00)
**Difficulties creating new and healthier habits**	40 (16.67)	58 (24.17)	84 (35.00)
**Difficulties avoiding unhealthy options**	34 (14.17)	56 (23.33)	84 (35.00)
**Difficulties creating new and more sustainable habits**	49 (20.42)	55 (22.92)	79 (32.92)
**Sustainable options are too limited**	59 (24.58)	19 (7.92)	48 (20.00)
**Lack of knowledge regarding more/less healthy**	30 (12.50)	23 (9.58)	45 (18.75)
**Lack of knowledge regarding more/less sustainable**	13 (5.42)	48 (20.00)	47 (19.58)
**No interest in eating healthier**	12 (5.00)	15 (6.25)	12 (5.00)
**No interest in eating taking into account the protection of the environment**	20 (8.33)	24 (10.00)	11 (4.58)
**Nothing, we already follow a healthy and sustainable diet**	32 (13.33)	18 (7.50)	32 (13.33)
**I don't know**	4 (1.67)	8 (3.33)	1 (0.42)

Note. Values are presented as n (%), based on 240 respondents per country.
